# A Tale of Immune Fragility: Severe Mycoplasma pneumoniae Infection With Mixed Autoimmune Hemolytic Anemia in Selective IgM Deficiency Disorder

**DOI:** 10.7759/cureus.93550

**Published:** 2025-09-30

**Authors:** Ramyashree Reddy, Samaga LN

**Affiliations:** 1 General Medicine, K.S. Hegde Medical Academy, Nitte University, Mangalore, IND

**Keywords:** atypical pneumonia, extrapulmonary manifestations, immune dysregulation, mixed autoimmune haemolytic anaemia, mycoplasma pneumoniae, selective igm deficiency

## Abstract

*Mycoplasma pneumoniae *is widely recognized as a leading cause of community-acquired atypical pneumonia and can trigger a wide spectrum of extra-pulmonary manifestations. This report discusses a rare clinical presentation of a young female patient who was admitted with an acute-onset, severe febrile illness. Her initial symptoms included a persistent cough with minimal purulent expectoration and mild difficulty in breathing, which rapidly progressed to severe dyspnea at rest. Her condition deteriorated swiftly, resulting in acute hypoxemic respiratory failure that required emergent endotracheal intubation and mechanical ventilation. Initial diagnostic imaging revealed diffuse bilateral interstitial and alveolar infiltrates, suggestive of severe pneumonia. The initial comprehensive workup, which included routine blood analyses, blood and sputum cultures, and a viral respiratory panel, was negative for any specific pathogen.

During the hospital stay, the patient developed new-onset moderate anemia. A detailed hematological investigation showed mixed autoimmune hemolytic anemia (AIHA). The presence of AIHA in a patient presenting with acute respiratory infection guided the diagnostic workup, ultimately identifying *M. pneumoniae* as the causative agent. Further immunological evaluation was done to explore this unusual presentation and revealed an underlying selective IgM deficiency with the presence of anti-La (SS-B) antibodies, suggesting a pre-existing state of immune dysregulation that may have predisposed her to these severe pulmonary and systemic manifestations.

The management of this case was multifaceted. The patient received aggressive respiratory support along with targeted antimicrobial therapy, an extended course of azithromycin. After a prolonged hospitalization, she was successfully weaned from mechanical ventilation and eventually made a full clinical recovery.

This report provides insight into the diverse clinical spectrum of *M. pneumoniae* infection and emphasizes the importance of maintaining a high index of suspicion in cases of unexplained AIHA. A clinical presentation of this severity in response to a common pathogen should prompt a thorough investigation for an underlying immunodeficiency state. This report is, to our knowledge, a rare clinical presentation that depicts the unique clinical triad of mixed AIHA, acute *M. pneumoniae* infection, and an underlying selective IgM deficiency.

## Introduction

*Mycoplasma pneumoniae* (*M. pneumoniae*), the smallest self-replicating bacterium, is primarily a mucosal pathogen that exhibits a clinical phenotype ranging from respiratory manifestations to systemic and autoimmune sequelae [1]. Fulminant pneumonia is a recognized progression of illness in approximately 2% of patients and is hypothesized to be a state of immune overactivity and a delayed hypersensitivity reaction [2].

While *M. pneumoniae* has been linked to autoimmune phenomena, such as cold autoimmune hemolytic anemia (AIHA), through IgM autoantibodies, other autoimmune responses, particularly in immunocompromised individuals, are less well understood. Cold AIHA typically occurs during the second or third week of illness and ranges from mild to severe, requiring transfusions [3]. Warm AIHA is secondary to IgG antibodies that bind to erythrocytes and are destroyed in the reticuloendothelial system [4]. The incidence of warm AIHA in *M. pneumoniae* infection has been seldom documented and has only been reported in instances of recurrent infections [5].

The propensity of *M. pneumoniae* to trigger autoimmune responses is established, yet its impact on the clinical trajectory in immunodeficient patients remains a critical knowledge gap. Selective IgM deficiency (SIgMD) is characterized by serum IgM levels below two standard deviations (SDs) of the mean for age, with normal IgG and IgA levels, and normal T-cell numbers and function, after excluding secondary causes such as infections and protein-losing conditions [6]. The prevalence of SIgMD is low in the general population, with community health screening surveys indicating a prevalence of approximately 0.03% for “complete” SIgMD. In immunology and immunodeficiency clinics, the prevalence ranges from 0.07% to 2.1%. These figures represent laboratory-defined SIgMD [6]. SIgMD presents in adulthood as recurrent sinopulmonary or genitourinary infections [7]. The European Society for Immunodeficiencies (ESID) registry criteria require repeatedly reduced serum IgM levels (e.g., <2 SD below mean or <10% of controls), normal IgG/IgA, and the exclusion of secondary hypogammaglobulinemia [8]. The manifestation of *M. pneumoniae* infection and the patterns of immune dysregulation in individuals with SIgMD have not been reported in the literature. This case report aims to illuminate an uncommon presentation of *M. pneumoniae*-associated immune dysregulation in an adult with SIgMD, underscoring the potential for unique host-pathogen interactions in this vulnerable patient group.

## Case presentation

A 24-year-old female patient with no significant past medical history, a non-smoker with no recent travel history, presented to the hospital with an acute febrile illness, cough, and progressively worsening dyspnea of eight-day duration. The patient had been in her usual state of health eight days ago when she developed a sudden, progressive cough without any diurnal or postural variations. The cough was associated with scanty, yellow, purulent, and non-foul-smelling sputum. She noticed intermittent high-grade fever without chills a day later. The patient developed breathlessness two days after the cough began, which gradually progressed from Modified Medical Research Council grade one to grade four over eight days. The patient had consulted a local primary healthcare physician and received antitussives and antipyretics; however, she reported no improvement.

On arrival at the hospital, the patient was conscious and oriented to time, place, and person. However, she was tachycardic with a pulse rate of 126 beats per minute, which was regular in rhythm, and a blood pressure of 100/60 mmHg. The patient was observed to be tachypneic with a respiratory rate of 30 cycles per minute and an oxygen saturation of 89% at ambient air. The patient was initiated on supplemental oxygen. A clinical evaluation began after obtaining the patient’s consent. Pulmonary examination revealed excessive usage of the accessory muscles of respiration. On palpation, there was decreased chest movement over the right lower lung field with an increased vocal fremitus. Woody dullness was noted on percussion over the lower right lung fields. On auscultation, bronchial breath sounds and coarse crepitations were observed throughout the right lower lung field, with increased vocal resonance. Cardiovascular, abdominal, and neurological examinations were unremarkable. No skin rashes were noted. Laboratory investigations revealed mild leukocytosis (WBC count: 11,060 cells/μL) and transaminitis (aspartate aminotransferase (AST): 110 U/L, alanine aminotransferase (ALT): 94 U/L), with elevated C-reactive protein (155.11 mg/L) and procalcitonin (6.94 ng/mL). The renal function test and urinary examination were relatively normal (Table 1). An infectious disease workup was done for dengue fever, malaria, leptospirosis, and rickettsial infection on day eight of the illness, which was negative. Blood and sputum cultures were sterile. Sputum Gram stain did not yield any respiratory pathogens. Fungal cultures and a KOH mount were performed to evaluate a fungal cause of bronchopneumonia; however, both tests were negative. H1N1 and H3N2 nasopharyngeal swabs were taken, which were negative. The COVID-19 polymerase chain reaction (PCR) test sent on day eight of the illness was negative (Table 2). A detailed chronological overview of the diagnostic workup, from initial presentation to the identification of etiology, is presented (Table 3).

**Table 1:** Laboratory analysis results with normal ranges. INR: international normalized ratio; RBC: red blood cell; AST: aspartate transaminase; ALT: alanine transaminase; ALP: alkaline phosphatase; CRP: C-reactive protein

Laboratory tests	Reference range	On admission	During second week	On discharge
Peripheral blood and urine tests				
Hemoglobin	12.0-15.0 g/dL	11.1 g/dL	7.1 g/dL	7.8 g/dL
Total leukocyte count (TLC)	4,000-10,000 cells/μL	11,060 cells/μL	11,000 cells/μL	4,920 cells/μL
Differential count (DC)				
Neutrophils	40%-80%	95.3%	72.7%	68.9%
Lymphocytes	20%-40%	3.5%	14%	19.4%
Monocytes	2%-10%	1%	8.8%	10.6%
Erythrocyte sedimentation rate (ESR)	0-20 mm/hr	27 mm/hr	52 mm/hr	-
Platelet count	1.5 × 10^5^/μL-4.5 × 10^5^/μL	1.6 × 10^5^/μL	3.1 × 10^5^/μL	3.8 × 10^5^/μL
Prothrombin time (sec)	Test: 13.29 s	16.1 s	16.5 s	16.1 s
INR	-	1.25	1.28	1.25
Peripheral smear	-	Normocytic normochromic anemia with neutrophilia	Dimorphic anemia with neutrophilia and clumped RBCs	Microspherocytes noted with dimorphic anemia
Urine routine	-	Normal	-	-
Serum biochemical test				
Random blood glucose	70-140 mg/dL	193 mg/dL	-	-
Albumin	3.5-5 g/dL	2.9 g/dL	2.5 g/dL	2.6 g/dL
Total bilirubin	0.2-1.3 mg/dL	0.41 mg/dL	0.64 mg/dL	0.81 mg/dL
Direct bilirubin	0-0.4 mg/dL	0.25 mg/dL	0.23 mg/dL	0.49 mg/dL
Indirect bilirubin	0-0.75 mg/dL	0.16 mg/dL	0.41 mg/dL	0.36 mg/dL
AST	5-30 U/L	110 U/L	83 U/L	49 U/L
ALT	4-36 U/L	94 U/L	75 U/L	64 U/L
ALP	38.0-126.0 U/L	82 U/L	80 U/L	88 U/L
Urea	16.6-48.5 mg/dL	12 mg/dL	13 mg/dL	13 mg/dL
Creatinine	0.7-1.4 mg/dL	0.6 mg/dL	0.6 mg/dL	0.5 mg/dL
Sodium	136-145 mmol/L	142 mmol/L	132 mmol/L	133 mmol/L
Potassium	3.5-5 mmol/L	3.6 mmol/L	4 mmol/L	4 mmol/L
Procalcitonin	<0.5 ng/mL	6.94 ng/mL	1 ng/mL	0.345 ng/mL
CRP	<6 mg/L	155.11 mg/L	65.1 mg/L	58.1 mg/L
D-dimer	<0.5 μg FEU/mL	>8 μg FEU/mL	-	-

**Table 2:** Microbiological evaluation. RT-PCR: reverse transcription polymerase chain reaction

Microbiological tests	Results
Dengue card test	Non-reactive
Malarial parasite (fluorescent) test	Negative
*Leptospira* card test	Negative
H3N2/H1N1 influenza A virus RT-PCR	Negative
Scrub typhus IgM antibody assay	Negative (1.763 units)
COVID RT-PCR	Negative
PCR for *Mycobacterium tuberculosis*	Not detected
Fungal KOH mount	No fungal elements
Blood culture & sensitivity	No growth on 5 days of incubation
Fungal culture	No fungal growth seen
HIV serology (chemiluminescent assay)	Non-reactive
Hepatitis C serology (chemiluminescent assay)	Non-reactive
*Mycoplasma* IgM	Positive

**Table 3:** Timeline of diagnostic tests from symptom onset with results. RT-PCR: reverse transcription polymerase chain reaction; LDH: lactate dehydrogenase; ANA: anti-nuclear antibody

Days from symptom onset	Tests	Results
Day 8	Dengue card test, malaria parasite fluorescent test, *Leptospira *IgM, H1N1, H3N2 swab, scrub typhus IgM, sputum Gram stain and cultures, COVID RT-PCR, PCR for *Mycobacterium tuberculosis*, blood culture & sensitivity	Negative
Day 11	Fungal KOH mount, fungal culture	Negative
Day 16	Peripheral smear, direct Coombs test, indirect Coombs test, LDH	Results suggestive of autoimmune hemolysis
Day 17	ANA screening and profile	Anti-SS-B/La positive
Day 18	Hepatitis C serology, HIV serology	Negative
Day 19	*Mycoplasma pneumoniae* IgM	Positive

**Figure 1:**
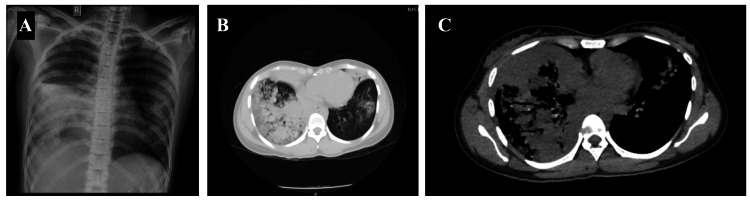
Radiological findings on admission. (A) Initial chest radiograph obtained on admission (day eight of illness) showing homogeneous opacity in the right mid and lower zones silhouetting the right cardiac border (silhouette sign), right costophrenic recess, and right hemidiaphragm without any tracheal or mediastinal shift. Multiple non-homogeneous opacities noted in the left mid and lower lung zones. (B) High-resolution computed tomography of the chest showing large areas of lobar consolidation involving the right middle lobe, superior and basal segments of the right lower lobe with air bronchogram, and a few patchy areas of consolidation in both lungs. (C) High-resolution computed tomography of the chest (mediastinal window) depicting mediastinal lymphadenopathy.

Arterial blood gas analysis revealed hypoxemic respiratory failure with compensated metabolic acidosis. ECG revealed sinus tachycardia, and a transthoracic echocardiogram was done, which was normal. Chest radiograph showed a homogeneous opacity in the right mid and lower zones silhouetting the right cardiac border, right costophrenic recess, and right hemidiaphragm without any tracheal or mediastinal shift (Figure 1A). Multiple non-homogeneous opacities were noted in the left mid and lower lung zones as well. High-resolution computed tomography (HRCT) of the chest depicted large areas of lobar consolidation involving the right middle lobe, superior segments, and all basal segments of the right lower lobe, with air bronchograms and a few patchy areas of consolidation in both lungs (Figure 1B). Mediastinal lymphadenopathy in the background of extensive consolidation suggested an infective etiology (Figure 1C). Despite empiric antibiotic therapy initiated on the eighth day of illness with ceftriaxone 1 g intravenous (IV) daily, the patient’s condition deteriorated, necessitating mechanical ventilation. The patient was intubated on day 12 of the illness and ventilated on synchronized intermittent mandatory ventilation (SIMV) mode with a fraction of inspired oxygen (FIO_2_) of 60% and positive end-expiratory pressure (PEEP) of 5 cm H_2_O. Arterial blood gas analysis showed a PaO₂ of 55 mmHg on FiO₂ 60%, yielding a PaO₂/FiO₂ ratio of approximately 92, suggestive of severe acute respiratory distress syndrome. Antibiotics were escalated to meropenem 1 g thrice daily on the 12th day of the illness because of worsening infection.

**Figure 2:**
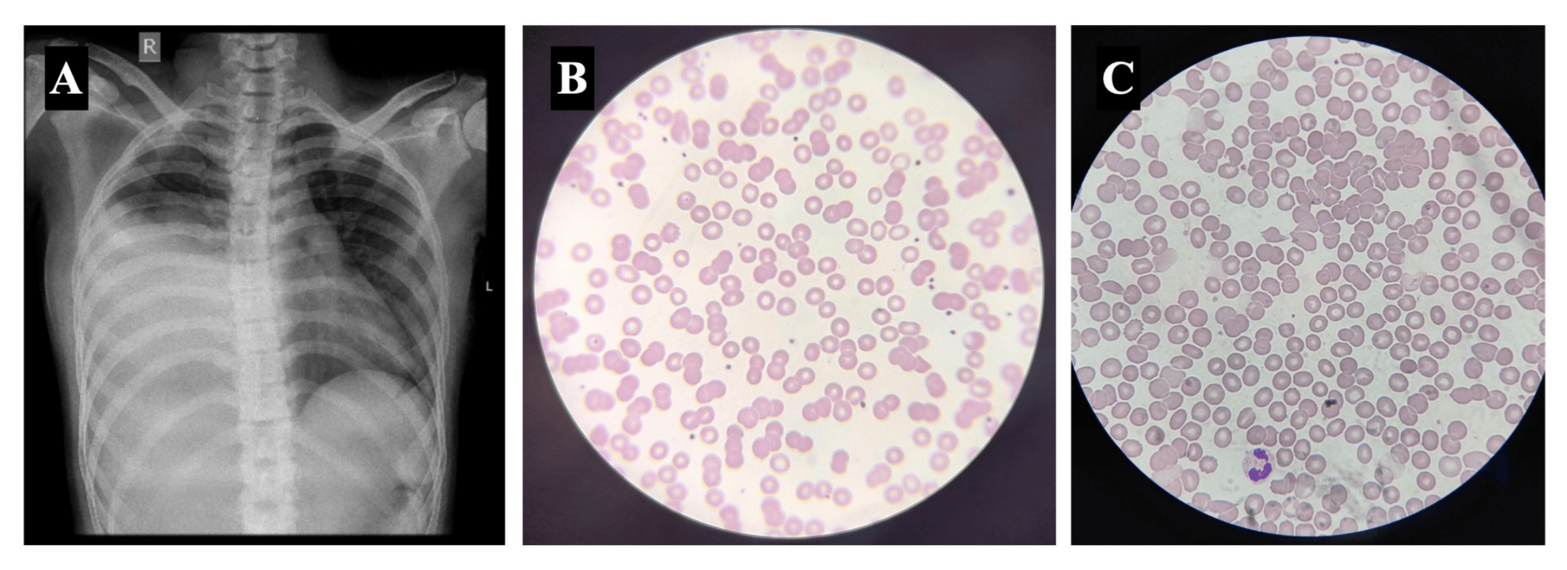
Chest radiograph and peripheral blood smear findings throughout the clinical course. (A) Chest radiograph obtained during the second week of illness showing progressively increasing opacity in the right mid and lower zones. (B) Peripheral blood smear during admission showing normocytic normochromic anemia. (C) Peripheral blood smear during the second week of illness showing clumping of RBC, suggestive of hemolysis. RBC: red blood cell

The patient developed a gradual drop in hemoglobin and grew progressively anemic during the second week of the illness. The initial hemoglobin on admission was 11.1 g/dL. By the 10th day of the illness, it had decreased to 10.1 g/dL. Subsequently, a sharper drop was noted over the following days from 10.1 to 7.8 g/dL by day 14, followed by a further decrease to 7.6 g/dL later on day 14. On day 15, hemoglobin fell to 7.2 g/dL, and by day 16, it reached 7.1 g/dL. Hemodynamic parameters remained stable during this period. A hematology consultation was sought to guide management. A workup for the new-onset worsening of anemia was sent (Table 4). With an elevated lactate dehydrogenase (LDH: 755 U/L) and positive direct Coombs test, a diagnosis of AIHA was suspected. Repeat chest radiograph during the second week showed worsening of opacities (Figure 2A). The peripheral blood smear on admission (Figure 2B) did not reveal any signs of hemolysis; however, a repeat peripheral blood smear showed clumping of erythrocytes (Figure 2C), and an extended blood group panel observed autoantibodies coating erythrocytes with in vivo and in vitro sensitization of red blood cells. Warm and cold hemagglutinins were noted in peripheral blood, confirming the presence of a mixed AIHA.

**Table 4:** Evaluation of hemolysis and immune dysregulation. ANA: anti-nuclear antibody

Hemolysis and autoimmune markers	Reference range	Results
Lactate dehydrogenase	120.0-246.0 U/L	755 U/L
Direct Coombs test (DCT)	-	Positive (complement-coated cells positive)
Indirect Coombs test	-	Positive
Cold antibodies	-	Positive
Warm antibodies	-	Present
ANA screening	-	1:80 titers, nuclear speckled pattern
ANA profile	-	Anti-SS-B/La positive
Total IgG	700-1,600 mg/dL	1,214 mg/dL
Total IgM	40-230 mg/dL	8 mg/dL
Total IgA	70-400 mg/dL	160 mg/dL

The hematology team recommended close monitoring of hemoglobin levels and emphasized treating the underlying infection as a priority. Given the severity of the infection, immunomodulatory therapies such as steroids, IV immunoglobulin (IVIG), and plasmapheresis were deferred due to potential risks. Transfusion triggers were carefully weighed against the patient's hemodynamic stability and clinical condition; no transfusion was initiated as the patient remained hemodynamically stable without signs of active bleeding or organ ischemia.

An underlying autoimmune pathology was suspected, and anti-nuclear antibody (ANA) screening and profile were done, which showed 1:80 titers with a nuclear speckled pattern positive for anti-SS-B/La antibodies. A comprehensive evaluation excluded common infectious triggers of AIHA. Hepatitis C and HIV were ruled out through serological analysis.

The serological diagnosis was made when *M. pneumoniae* IgM was detected positive. *M. pneumoniae* IgM assay was performed on day 19 of the illness using a chemiluminescent immunoassay, with a cut-off value of 10 index. Results below 10 were considered negative, and results above 10 were considered positive. In our patient, the IgM titer was 35.20 index. The patient was initiated on a course of oral azithromycin 500 mg daily on day 19 of the illness, which yielded a remarkable improvement in oxygenation status and general condition by the end of the second week. Mechanical ventilation and weaning steps were performed according to established protocols. Daily assessments, including readiness screens and spontaneous breathing trials, guided ventilator weaning without complications. The patient was successfully extubated on day 22 of the illness. The patient tolerated extubation well, and there were no ventilator-associated complications noted during her course.

A workup for an underlying immunodeficiency disorder responsible for a severe course of pneumonia was done, which yielded low levels of IgM (IgM: 8 mg/dL) with normal levels of IgA and IgG. Once low levels of IgM were detected, other causes such as proteinuria, protein-losing states, and exposure to immunosuppressant therapy were excluded. Following the exclusion of these conditions, a diagnosis of SIgMD disorder was made (Table 4). The patient resumed normal functionality over the next few days and was transferred to the wards for further recovery. Azithromycin was continued for 21 days due to the severity of the infection. This treatment approach was guided by the patient's clinical response and aimed to ensure comprehensive eradication of the pathogen while supporting the resolution of immune-mediated complications. The patient was monitored for a week with regular evaluation of hemoglobin and chest radiograph.

**Figure 3:**
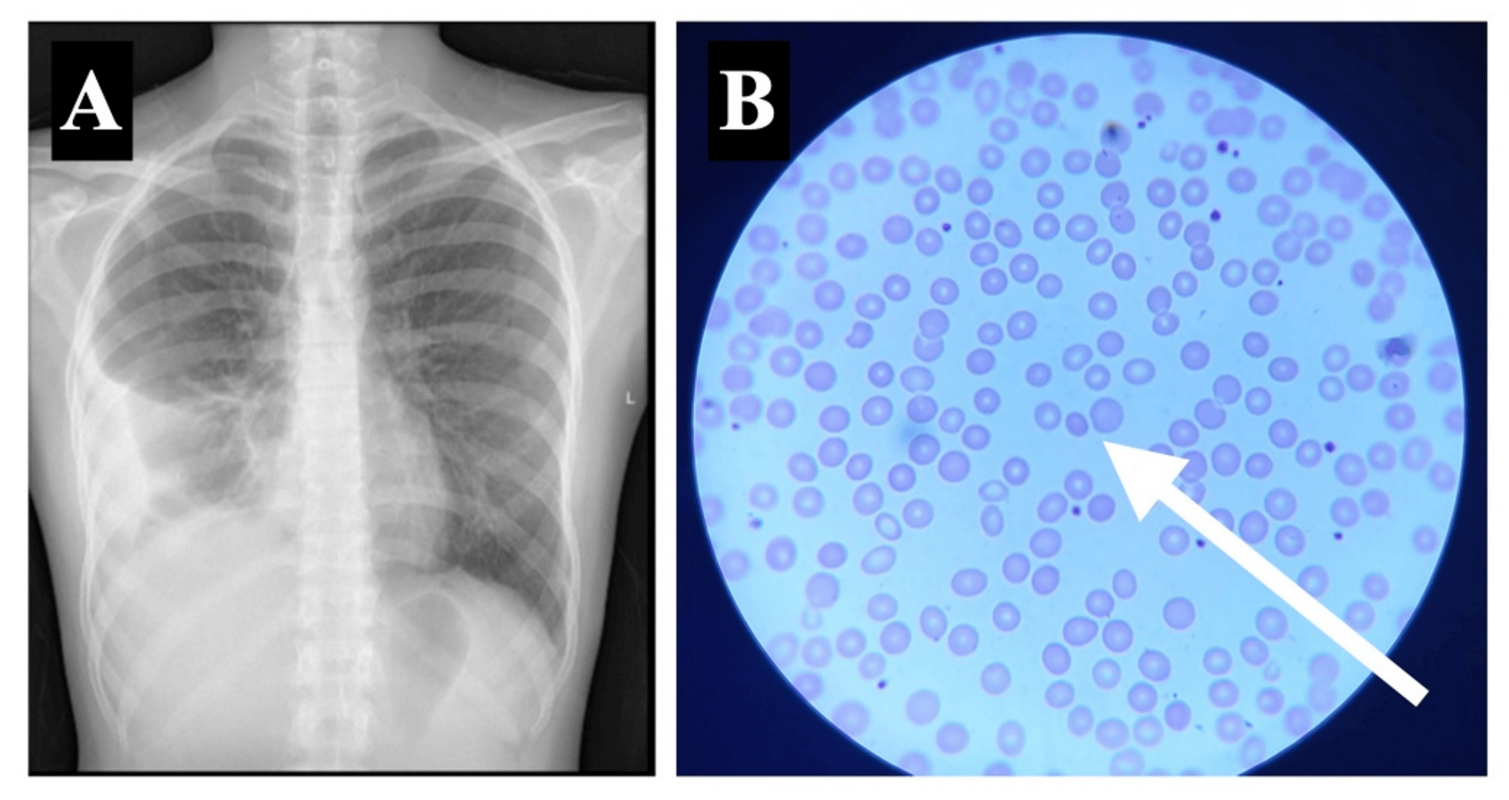
Chest radiograph and peripheral blood smear findings during discharge. (A) Chest radiograph obtained during discharge showing partial resolution of infiltrates. (B) Peripheral blood smear obtained on discharge showing microspherocyte (white arrow) and reduced agglutination of RBC. RBC: red blood cell

The patient was discharged following clinical improvement and advised to follow up under the care of an immunologist and adhere to vaccinations during subsequent visits. A chest radiograph performed upon discharge revealed significant improvement, though not complete resolution, of the previously observed opacities (Figure 3A). The peripheral smear on discharge showed a reduction in hemolysis with a few microspherocytes (Figure 3B).

## Discussion

This case describes a unique presentation of severe *M. pneumoniae* infection complicated by mixed AIHA in a patient subsequently diagnosed with underlying SIgMD. *M. pneumoniae* predominantly causes mild respiratory infections by adhering to the respiratory epithelium through specialized adhesion proteins and inducing cellular injury via the production of reactive oxygen species [9]. *M. pneumoniae* is frequently associated with extra-pulmonary features mediated by direct toxicity to cells or through the formation of immune complexes [10].

This specific triad of severe *M. pneumoniae* with mixed AIHA in an adult patient with SIgMD highlights a unique interaction of infection, autoimmunity, and primary immunodeficiency. AIHA may be broadly classified as warm AIHA, cold AIHA, and mixed AIHA based on the type of agglutinins present in peripheral blood [11]. While *Mycoplasma* has been associated with high titers of cold hemagglutinins, there have been a few case reports with concurrent *M. pneumoniae* infection and warm AIHA. Cassimos et al. described recurrent episodes of *Mycoplasma* infection inducing a state of warm AIHA in a pediatric patient with Down syndrome [5]. Existing reports of *Mycoplasma*-associated warm AIHA often describe cases in pediatric populations or individuals with a background of recurrent infections, rather than an acute, de novo presentation in an adult with a newly diagnosed primary immunodeficiency [12]. The presentation of mixed AIHA, characterized by the presence of both warm and cold autoantibodies, directly associated with an acute *Mycoplasma* infection, is highly unusual and represents a significant diagnostic challenge.

The diagnostic evaluation in the presented case was intricate. The absence of common microbial pathogens on initial screening, along with definitive hematological evidence of AIHA, led to the consideration of *M. pneumoniae* as a potential cause. Subsequent evaluation of *Mycoplasma* IgM titers confirmed an acute infection. The unusual severity of the infection, combined with mixed AIHA, prompted further immunological assessment, which revealed isolated low levels of serum IgM, consistent with SIgMD disorder.

SIgMD is a rare primary immunodeficiency characterized by a serum IgM level below two SDs with normal levels of serum IgA, IgG [13]. SIgMD is not associated with any specific inheritance pattern but has been known to be associated with 22q11.2 deletion [13]. Clinically, SIgMD disorder manifests as recurrent infections, atopy or autoimmune disorders, and malignancy [13].

The pathobiological interplay between the *Mycoplasma* infection, the subsequent development of mixed AIHA, and the patient's underlying SIgMD is a central point of interest in the case. The patient's immunodeficiency likely predisposed them to severe illness and may have also induced immune overactivity, manifesting as autoimmunity. The SIgMD disorder could have promoted a broader B-cell activation and loss of tolerance, leading to the production of both warm and cold autoantibodies, culminating in mixed AIHA. Alternatively, the *Mycoplasma* infection in a host with compromised IgM-mediated immune surveillance due to SIgMD may have served as a potent immunological trigger, exacerbating a latent autoimmune state. The management of this patient required a multifaceted approach. Azithromycin was administered for three weeks to address the underlying *M. pneumoniae* infection. Although corticosteroids are typically indicated in the treatment of AIHA, they were withheld in this case due to the severity of the infection and the stability of serial hemoglobin levels. In contrast, Wandro et al. reported a case of warm AIHA in a pediatric patient who presented with severe anemia necessitating blood transfusions and IVIG therapy [12].

The patient was counseled extensively on the underlying immunodeficiency disorder and its implications, including the need for prophylactic vaccinations for primary prevention of diseases. The etiology of the autoimmune state observed in this patient remains unknown. The underlying cause of the autoimmune state, whether a transient response to the infection in a susceptible individual or the unmasking of an underlying autoimmune diathesis associated with SIgMD, remains uncertain and requires ongoing follow-up.

While the current study describes a novel and complex association, it cannot establish definitive causality between severe *Mycoplasma* infection, SIgMD, and the development of mixed AIHA, nor can the findings be generalized. However, this case significantly adds to the spectrum of autoimmune complications associated with *M. pneumoniae* infection. It underscores the importance for clinicians to consider *M. pneumoniae* in the differential diagnosis of AIHA, especially when atypical features are present, and to consider underlying immunodeficiency in cases of unusually severe or complex presentations of *M. pneumoniae* infection.

## Conclusions

This case report serves as a useful and practical reminder for clinicians to broaden their differential diagnosis when faced with a patient presenting with mixed AIHA, particularly when it occurs in association with a severe respiratory illness. Although the association between *M. pneumoniae* and cold agglutinin disease is well-established, its role in triggering mixed AIHA is uncommon and, therefore, can be easily overlooked. Therefore, in cases of AIHA that do not fit the usual clinical pattern, clinicians should keep a high index of suspicion for *M. pneumoniae* as the etiological agent, particularly when respiratory symptoms are prominent and initial standard antibiotic therapies prove ineffective. Furthermore, the severity of the patient's pneumonia and the unusual autoimmune sequelae should prompt a deeper investigation into the patient's underlying immune status. When a common pathogen like *M. pneumoniae* leads to a life-threatening illness or precipitates a complex autoimmune reaction, it may be a sign of a previously undiagnosed primary immunodeficiency. As this case demonstrates, the discovery of SIgMD was pivotal in understanding the patient's vulnerability. Ignoring this possibility may lead to missed opportunities for appropriate long-term management, including prophylactic measures and vaccinations, which are essential for preventing future life-threatening events in these susceptible individuals.

It is important to acknowledge the limitations within the evaluation of the case. Firstly, the immunohematology tests, including the direct antiglobulin test (DAT) and cold agglutinin testing, were qualitative rather than quantitative. This precluded the determination of specific titers (e.g., cold and warm agglutinin titer) and detailed subclass distinctions, such as IgG and/or C3d positivity. While this reflects resource constraints in certain clinical settings, the qualitative results, alongside clinical findings and other laboratory parameters, were deemed sufficient for the diagnosis of mixed AIHA. Secondly, a limitation in the diagnostic workup for *M. pneumoniae* was the absence of *Mycoplasma* IgG testing and PCR. Given the acute presentation of the infection, IgG evaluation was not deemed necessary. However, PCR would have been a more suitable diagnostic option had the evaluation been sent in the first week of illness. Furthermore, the diagnosis relied on a single-sample serology for *Mycoplasma* IgM, which carries inherent limitations, including the potential for false positives due to cross-reactivity and the inability to definitively distinguish between recent and past infections. This lack of serial IgM testing and comprehensive AIHA evaluation, due to clinical constraints, means that a full longitudinal assessment could not be performed. Ultimately, this case contributes significantly to the existing medical literature by meticulously documenting a complex triad of severe *M. pneumoniae* infection, mixed AIHA, and SIgMD in an adult. Reporting this unusual association creates a greater awareness among healthcare professionals of the broad and unpredictable clinical spectrum of *M. pneumoniae* infections. The report highlights that this common bacterium has the potential to provoke immune dysregulation, leading to severe and multifaceted clinical presentations, especially in individuals with compromised immune systems. This enhanced understanding is vital for improving diagnostic accuracy, guiding appropriate therapeutic strategies, and ultimately, improving outcomes for patients with these challenging and atypical presentations.
